# Inter- and intrapopulation resource use variation of marine subsidized western fence lizards

**DOI:** 10.1007/s00442-023-05496-6

**Published:** 2024-01-20

**Authors:** Alexi Ebersole, Marie E. Bunker, Stacey L. Weiss, Kena Fox-Dobbs

**Affiliations:** 1 Department of Biology, University of Puget Sound, Tacoma, WA 98416 USA; 2 Department of Geology, University of Puget Sound, Tacoma, WA 98416 USA

**Keywords:** Interpopulation specialization, Marine resource subsidy, Isotopic niche, Stable isotope ecology, Transitional landscapes

## Abstract

Marine resource subsidies alter consumer dynamics of recipient populations in coastal systems. The response to these subsidies by generalist consumers is often not uniform, creating inter- and intrapopulation diet variation and niche diversification that may be intensified across heterogeneous landscapes. We sampled western fence lizards, *Sceloporus occidentalis*, from Puget Sound beaches and coastal and inland forest habitats, in addition to the lizards’ marine and terrestrial prey items to quantify marine and terrestrial resource use with stable isotope analysis and mixing models. Beach lizards had higher average δ^13^C and δ^15^N values compared to coastal and inland forest lizards, exhibiting a strong mixing line between marine and terrestrial prey items. Across five beach sites, lizard populations received 20–51% of their diet from marine resources, on average, with individual lizards ranging between 7 and 86% marine diet. The hillslope of the transition zone between marine and terrestrial environments at beach sites was positively associated with marine-based diets, as the steepest sloped beach sites had the highest percent marine diets. Within-beach variation in transition zone slope was positively correlated with the isotopic niche space of beach lizard populations. These results demonstrate that physiography of transitional landscapes can mediate resource flow between environments, and variable habitat topography promotes niche diversification within lizard populations. Marine resource subsidization of Puget Sound beach *S. occidentalis* populations may facilitate occupation of the northwesternmost edge of the species range. Shoreline restoration and driftwood beach habitat conservation are important to support the unique ecology of Puget Sound *S. occidentalis.*

## Introduction

Species with wide ranges are often generalist consumers suited for a large breadth of environmental conditions, but populations can be specialized to the local conditions of their habitat, such as prey availability, creating significant population-level differences (Brown 1995; Gregory & Isaac 2004; Obrist et al. 2022). In addition, individuals within a population can show unique resource partitioning and prey selection (Bolnick et al. 2003; Roughgarden 1972). Individual resource use within populations reflects niche variation and can be influenced by spatial subsidies that provide novel foraging opportunities through the exchange of resources from one ecosystem to another (Araújo et al. 2008, 2011; Polis et al. 1997; Polis & Winemiller 2013; Van Valen 1965). The intersection between marine and terrestrial ecosystems provides the opportunity for unique combinations of resource use by generalist populations, which may be explained by sex, age, morphology, or other finer-scale individual specializations (Bolnick et al. 2003).

The western fence lizard (*Sceloporus occidentalis*) is a wide-ranging species inhabiting diverse topography and climates from Baja, Mexico, north to the Puget Sound region in Washington, the USA, and as far inland as Utah (Bouzid et al. 2022). At the species’ northwestern range limit, *S. occidentalis* have unique life history traits compared to southern populations. For example, *S. occidentalis* in the Puget Sound region annually hibernate, have large clutch sizes, and have an interesting evolutionary history resulting in isolated, genetically distinct populations (Davis et al. 2022; Sinervo 1990; Tsuji 1988). Additionally, the distribution of fragmented *S. occidentalis* populations in the Puget Sound region is almost entirely restricted to patchy coastal habitats (Davis et al. 2022). The microclimate, habitat structure, and prey availability of the coastal Puget Sound environment could all be extending the range of *S. occidentalis* at their geographic extreme. This idea is supported by previous studies of coastal lizard populations that have observed higher lizard abundance and density compared to inland populations due to both marine and terrestrial prey availability (Barrett et al. 2005; Spiller et al. 2010).

The flow of marine energy and nutrients to terrestrial ecosystems via spatial subsidies has been well documented in many systems (Polis et al. 1997, 2004). Marine resource subsidies can be indirect, such as seabird guano or salmon carcass fertilization at the base of terrestrial food webs (Anderson and Polis 1999; Helfield and Naiman 2001), or direct, such as consumption of marine prey by terrestrial predators and scavengers (Chamberlain et al. 2005; Darimont and Reimchen 2002). Stable isotope analyses (SIA) are routinely used to investigate how resource subsidies link adjacent habitats and environments (Bartels et al. 2012; Pringle and Fox-Dobbs 2008). Carbon and nitrogen in consumer tissues are derived from diet, and therefore the isotopic composition (reported as δ^13^C and δ^15^N values) of predators such as lizards reflect the values of their prey (Boecklen et al. 2011; Peterson and Fry 1987; Warne et al. 2010; Warne and Wolf 2021). The substantial and predictable differences in marine versus terrestrial food web δ^13^C and δ^15^N values mean that SIA has been widely used for decades to make robust estimates of the relative contributions of diet sources from each environment for coastal consumers (Hobson and Sealy 1991). SIA has documented how coastal lizard communities partially depend on aquatic resource subsidies in pond ecosystems (Martins et al. 2021). And in marine systems, intertidal wrack (i.e., macroalgae) and arthropods can be a consistent marine resource subsidy, contributing to the diets of small nearshore terrestrial vertebrate predator food webs (Barrett et al. 2005; Spiller et al. 2010; Stapp and Polis 2003), including in the Pacific Northwest (Davidson et al. 2021). Several key variables control the deposition and accumulation of wrack on shorelines (e.g., donor habitat, transport, and shoreline physical properties) (Obrist et al. 2022; Wickham et al. 2020), which in turn may regulate the strength of the marine resource subsidy to terrestrial predators. Additionally, the flow of terrestrial prey to beach-dwelling predators may be influenced by factors, such as topographic gradients and terrestrial productivity.

Here we utilize SIA of lizards and their prey to study the role of marine resource use in beach-dwelling *S. occidentalis* lizard populations in Puget Sound. We apply statistical models to estimate the strength of a marine resource subsidy, and to calculate the isotopic niche area for five populations of beach-dwelling lizards. We demonstrate how geographically restricted the marine subsidy is by also analyzing lizards from forest habitats ranging from < 1000 m (coastal forest) to > 100 km (inland forest) from the Puget Sound beaches. We hypothesize that intertidal wrack-derived arthropod preys serve as a substantial marine subsidy in the diets of *S. occidentalis* populations on Puget Sound beaches and predict that, across beaches, utilization of marine resources by individual lizards will vary with beach topography and with demographic factors like sex, body size, and body condition (Bolnick et al. 2003). Finally, we predict that lizards from different habitats—beach, coastal forest, and inland forest—will have distinct distributions of δ^13^C and δ^15^N values that reflect the consumption of marine versus terrestrial arthropod prey and the influence of hydroclimate on terrestrial plant δ^13^C values at the coastal and inland forest habitats (Kohn 2010).

## Methods

### Study sites

**Fig. 1:**
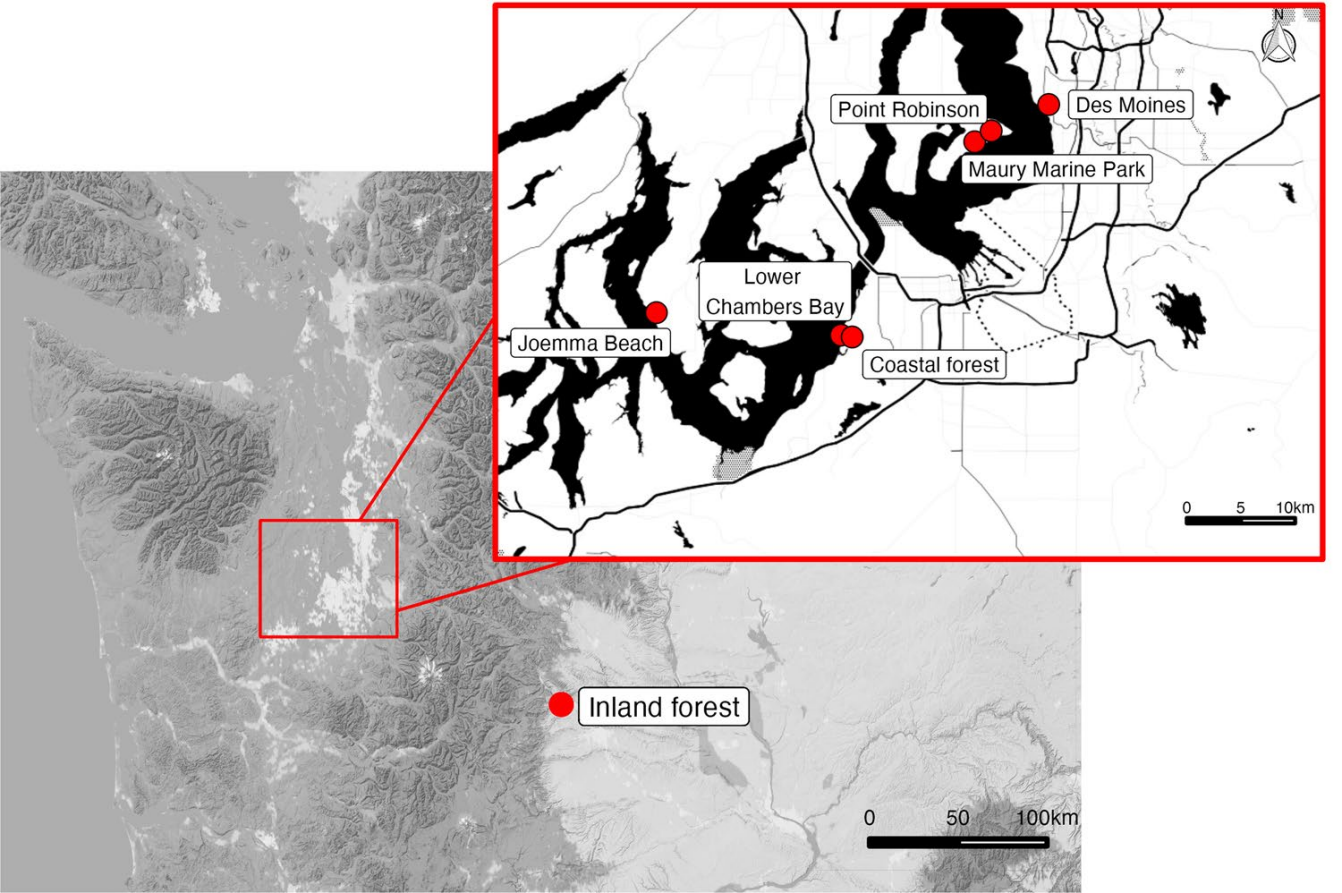
Map of *Sceloporus occidentalis* lizard study sites in Washington State. Inset shows the five beach study sites and the coastal forest site, located on the Puget Sound. The inland forest site was in Bear Canyon, Yakima County, WA. Map tiles by Stamen Design, under CC BY 3.0. Data by OpenStreetMap, under ODbL

We collected data from seven *S. occidentalis* populations across three unique habitats in Washington State, the USA. Study sites consisted of five South Puget Sound beach lizard populations, a coastal forest population, and an inland forest population (Fig. 1). The beach study sites [elevation = 0 m above sea level (m asl)] were all characterized by sand and gravel substrate, large driftwood logs at the upper tidal line, and southerly exposure. Beach study sites were: Des Moines (DM), Joemma Beach (JB), Lower Chambers Bay (LCB), Maury Marine Park (MMP), and Point Robinson (PR) (Fig. 1). The coastal forest study site (elevation = 70 m asl) was approximately 1000 m away from LCB, and was characterized by mature Douglas fir trees (*Pseudotsuga menziesii*), and an understory of predominantly salal (*Gaultheria shallon*) and blackberry (*Rubus armeniacus*). LCB and the coastal forest habitats’ high and low temperature average from May through August are 22.6 and 11.8 °C with 996 mm/yr precipitation (*Weather Averages, Washington, USA*, 2022). The inland forest study site (elevation = 650 m asl) was located in Bear Canyon, Oak Creek Wildlife Area, Yakima County, Washington, > 100 km from the Puget Sound beaches. The site followed a dry creek bed of cobbles and boulders, flanked by a primarily coniferous (*Pinus ponderosa, Pseudotsuga menziesii*) forest with a patchy understory of shrubs and grasses. Its high and low temperatures from May through August averaged 27.6 and 9.3 °C with 209 mm/yr precipitation (*Weather Averages, Washington, USA*, 2022).

### Sample collection

In May through August 2020 and 2021, we obtained samples from adult female and male *S. occidentalis* (*n* = 10 lizards at DM, 11 at JB, 10 at MMP, 9 at LCB, 7 at PR, 10 at coastal forest, and 11 at inland forest study sites, 68 lizards total; Table 1), captured opportunistically via a small string lasso at the end of a 2 m pole. For SIA, we removed a small portion of the tip of the lizard's tail and kept it on ice in the field for no longer than 5 h, and then stored the tissue sample at −80 °C until processing. Prior to release, we measured lizard snout to vent length (SVL) using a handheld ruler, and body mass using a Pesola spring scale. Body condition was calculated as the residuals of the regression (body mass) ~ (SVL^3^), as in Weiss (2006) and Weiss and Brower (2021), and is used as a metric of individual fat storage. To avoid recapture, we toe-clipped lizards for permanent identification, and applied a small paint mark on the lizards’ back. Capture location coordinates were recorded using a handheld GPS unit. All research activities were conducted under compliance with the University of Puget Sound Institutional Animal Care and Use Committee (PS18002 and PS21003) and the Washington Department of Fish and Wildlife (21-002b and 20-039).

Arthropod and plant samples were collected for SIA and beach lizard diet reconstruction. The LCB beach site was considered to be representative of all beach sites due to close geographic proximity (maximum distance ~ 25 km) and environmental similarity. Terrestrial arthropods were collected by sweep-net sampling of understory vegetation and ground level pitfall traps at the LCB beach site as well as at the inland and coastal forest sites. Intertidal arthropods (Talitridae) were collected at LCB by hand directly from stranded macro-algae along the tidal line. Representative samples of arthropods across taxa (Coleoptera, Hymenoptera, Araneae, Orthoptera, and Isopoda), body size, and trophic level (*n* = 17 arthropods) were selected for isotopic analysis. For plant samples at LCB, we collected Himalayan blackberry leaves and beach-stranded macro-algae fragments. Each sample was a composite of three nearby plants or algae clumps. At the inland site, we collected foliage of seven abundant grass taxa to confirm the absence of C_4_ vegetation. Like lizard tissue samples, arthropods and plant samples were stored on ice in the field for no more than 8 h, then stored at −80 °C until processing.

### Sample preparation and isotopic analyses

Lizard tail tissue samples were prepared and analyzed following the methods in Pringle et al. (2019). Tail samples were dried to a constant weight at 40 °C, and then wiped with 70% ethanol to remove surface contamination. We used elemental carbon and nitrogen weight percent ratios (C:N) to verify that lizard tail samples (a composite of scale keratin, skin, and proteinaceous connective tissue) were comparable among individuals. The tail C:N ratios were consistent (3.1–3.9 range, except for 6 samples with slightly higher ratios, but these samples were not isotopically anomalous and thus retained for analysis).

For arthropods, we targeted legs and head capsules for stable isotope analysis, and samples were prepared following the methods of Pringle and Fox-Dobbs (2008). If arthropods were too small to be analyzed individually, samples consisted of body parts from multiple individuals of the same morpho-species. The arthropod weight percent C:N ratios ranged from 3.7 to 5.9 confirming that all samples were a composite of proteinaceous and chitin tissues.

Approximately 1 mg of lizard tail and arthropod samples were weighed into tin boats for SIA. Plant and algae samples were dried at 40 °C, homogenized with mortar and pestle, and approximately 5 mg samples were weighed into tin boats. Samples were analyzed at the University of Washington Stable Isotope Laboratory, and the University of Colorado Boulder Earth Systems Stable Isotope Laboratory. Stable isotope compositions for all sample types (lizard tail tissue, arthropod, and plant) are reported using the δ notation and referenced to Vienna PeeDee Belemnite for carbon, and air N_2_ for nitrogen.

### Diet and isotopic niche modeling for beach lizards

In R (Version 4.2.1), we used the MixSIAR package to model the diets of individual lizards in the beach sites, and to estimate the relative percentages of marine and terrestrial arthropod prey sources in diet (R Core Team 2021; Stock et al. 2018; Stock and Semmens 2016). For the MixSIAR model, we used published diet to tissue discrimination values, Δ^13^C_tissue-diet_ and Δ^15^N_tissue-diet_, for small-bodied insectivorous lizards. Since the tail tip samples were a composite by biomass of primarily scale (keratin), skin, and connective tissue, we relied on Δ^13^C_tissue-diet_ and Δ^15^N_tissue-diet_ values calculated for lizard claw keratin (+ 1.2 ± 0.4‰, and + 0.7 ± 0.3‰, respectively) (Lattanzio and Miles 2016), and a Δ^13^C_tissue-diet_ value for lizard skin (−0.8 ± 0.5‰) (Warne et al. 2010). A comparable Δ^15^N_tissue-diet_ value for lizard skin was not published since the tissue did not reach equilibrium by the end of the 360 day study (Warne and Wolf 2021). These studies highlight the relatively slow carbon and nitrogen incorporation rates measured in terrestrial ectotherm tissues, and suggest that our samples likely represent an average diet over months (Warne et al. 2010; Warne and Wolf 2021). We modeled the beach-dwelling lizard diet twice with MixSIAR, once each using claw and skin Δ^13^C_tissue-diet_ values, and claw Δ^15^N_tissue-diet_ value held constant in both. The ecological relevance of the diet estimates from the two models was similar. We also independently estimated the percentage of marine diet for beach lizards by calculating an alpha value (Spiller et al. 2010),

$$\alpha = \left\{\left[\left(\delta^{13} C_{{\rm consumer}} -\Delta^{13} C\right)-\delta^{13} C_{{\rm terrestrial}}\right]/\left(\delta ^{13} C_{{\rm marine}} -\delta ^{13} C_{{\rm terrestrial}} \right)\right\}*100$$

where δ^13^C_consumer_ is the lizard δ^13^C value, δ^13^C_terrestrial_ is the average blackberry value (−30.6‰), and δ^13^C_marine_ is the average algae value (−11.4‰). We did not adopt the approach of Post (2002) which assumes no diet to tissue discrimination value, but instead followed Spiller et al. (2010), and used a Δ^13^C_tissue-diet_ value of 3.8‰ for terrestrial prey (measured from plants and herbivorous insects), and no fractionation for marine prey and lizards. We subsequently compared the results of the MixSIAR and α value approaches for estimating marine contribution to lizard diet. We present the proportion marine diet estimates for all individual beach lizards from the two MixSIAR models, and the α value equation in the publicly archived data sheet for direct comparison.

We estimated and compared the isotopic niche space of the beach lizard populations using the standard ellipse area corrected for small sample sizes (SEA_C_), with the Stable Isotope Bayesian Ellipses in R (SIBER) package (Jackson et al. 2011; R Core Team 2021). Bayesian approaches incorporate sampling biases and smaller sample sizes, making them advantageous for estimating niche metrics. SIBER repeatedly assigns measures of uncertainty, in this case based on Markov-Chain Monte Carlo simulation, to construct SEA_C_ (Jackson et al. 2011).

### Transition zone slope analyses

We examined whether the slope of the transition zone between marine and terrestrial environments (e.g., unvegetated bluff, open meadow, and coastal forest) influenced marine versus terrestrial resource use and isotopic niche space of beach lizard populations. For each beach, we used Google Earth satellite images (Google Earth Pro 2022) to calculate the slope as the elevation relief along five 75 m transects that extended inland perpendicular from sea level (0 m asl) along the shoreline where lizards were captured. We then calculated the average and standard deviation of the transition zone slope for each beach site.

### Statistical analyses

We examined how δ^13^C and δ^15^N values varied across our three habitats using Kruskal–Wallis followed by Dunnett’s tests. We used regression to determine how SEA_C_ and percent marine diet were affected by aspects of beach topography. We used a *t*-test to determine whether the percent marine diet (log-transformed to meet assumptions of normality) differed between males and females, and we used correlation to examine its relationship to lizard body condition, size, and mass. All figures were generated using the ggplot2 and ggpubr packages (Wickham 2016; Kassambra 2023) and maps were generated using the ggmap package (Wickham and Kahle 2013).

## Results

### Variation in lizard δ^13^C and δ^15^N values across habitat types

**Fig. 2:**
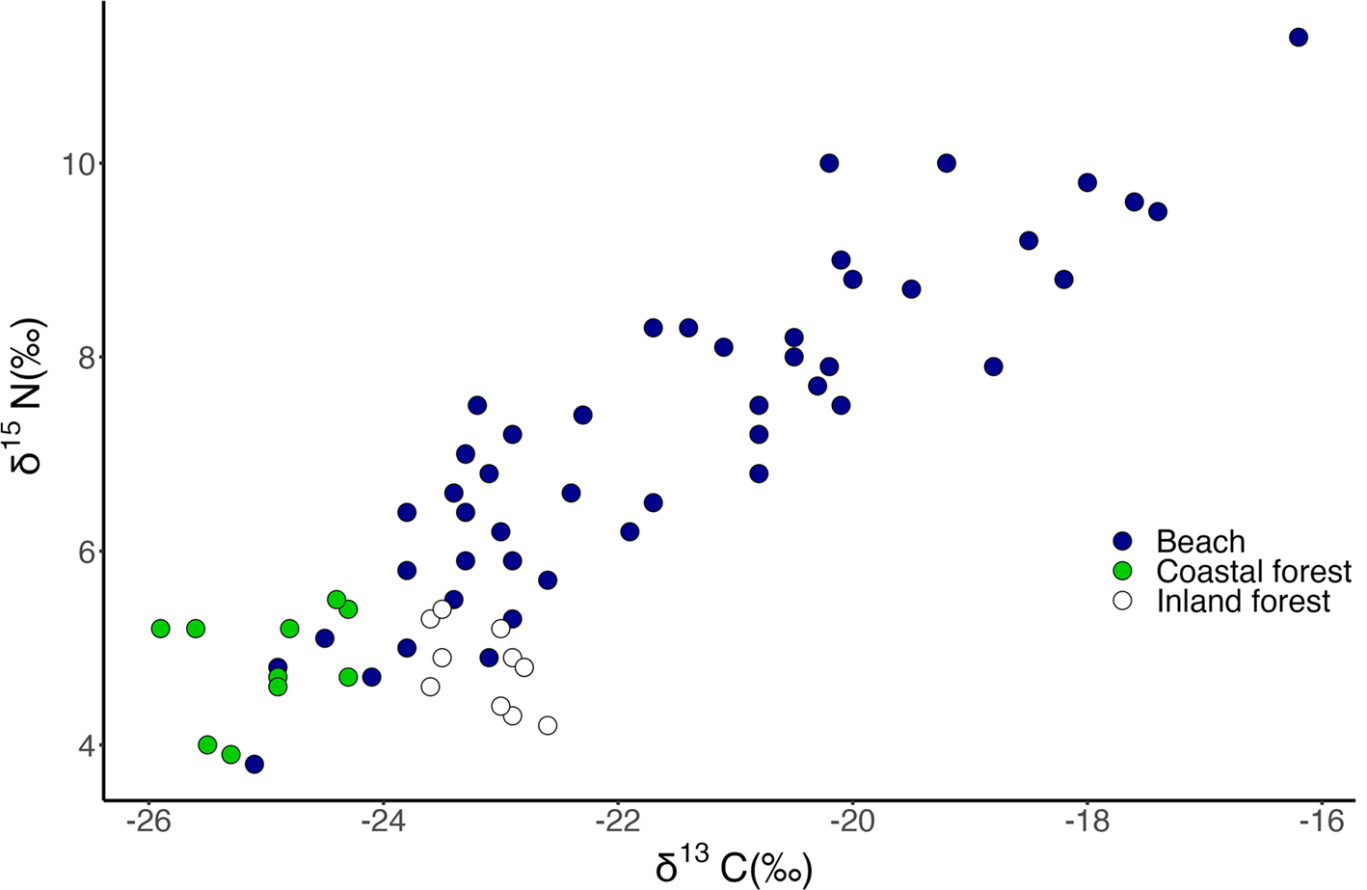
δ^13^C and δ^15^N values of *S. occidentalis* lizards at three habitats in Washington State: Puget Sound beaches, coastal forest, and inland forest

*S. occidentalis* lizards from the beach, coastal forest, and inland forest habitats occupied different δ^13^C and δ^15^N bivariate isospace (δ^13^C: χ^2^ = 25.99, df = 2, *P* << 0.001; δ^15^N: χ^2^ = 31.05, df = 2, *P* << 0.001; Fig. 2). Beach lizard mean δ^13^C and δ^15^N values were higher than those from the other two study habitats (δ^13^C: beach-coastal *P* << 0.001, beach–inland *P* = 0.022; δ^15^N: beach-coastal *P* << 0.001, beach–inland *P* << 0.001). The beach lizard isotopic values were also substantially more variable than isotopic values from either of the forest habitats; the range in both δ^13^C and δ^15^N values of beach lizards was approximately 10‰, compared to maximum ranges of 1.6‰ in δ^13^C and δ^15^N values for lizards in the two forest habitats. The ranges in δ^13^C and δ^15^N values at individual beach sites were also greater (1.5× to 4×) than the ranges for the forest habitats.

### Variation in lizard δ^13^C and δ^15^N values across-beach sites

**Fig. 3:**
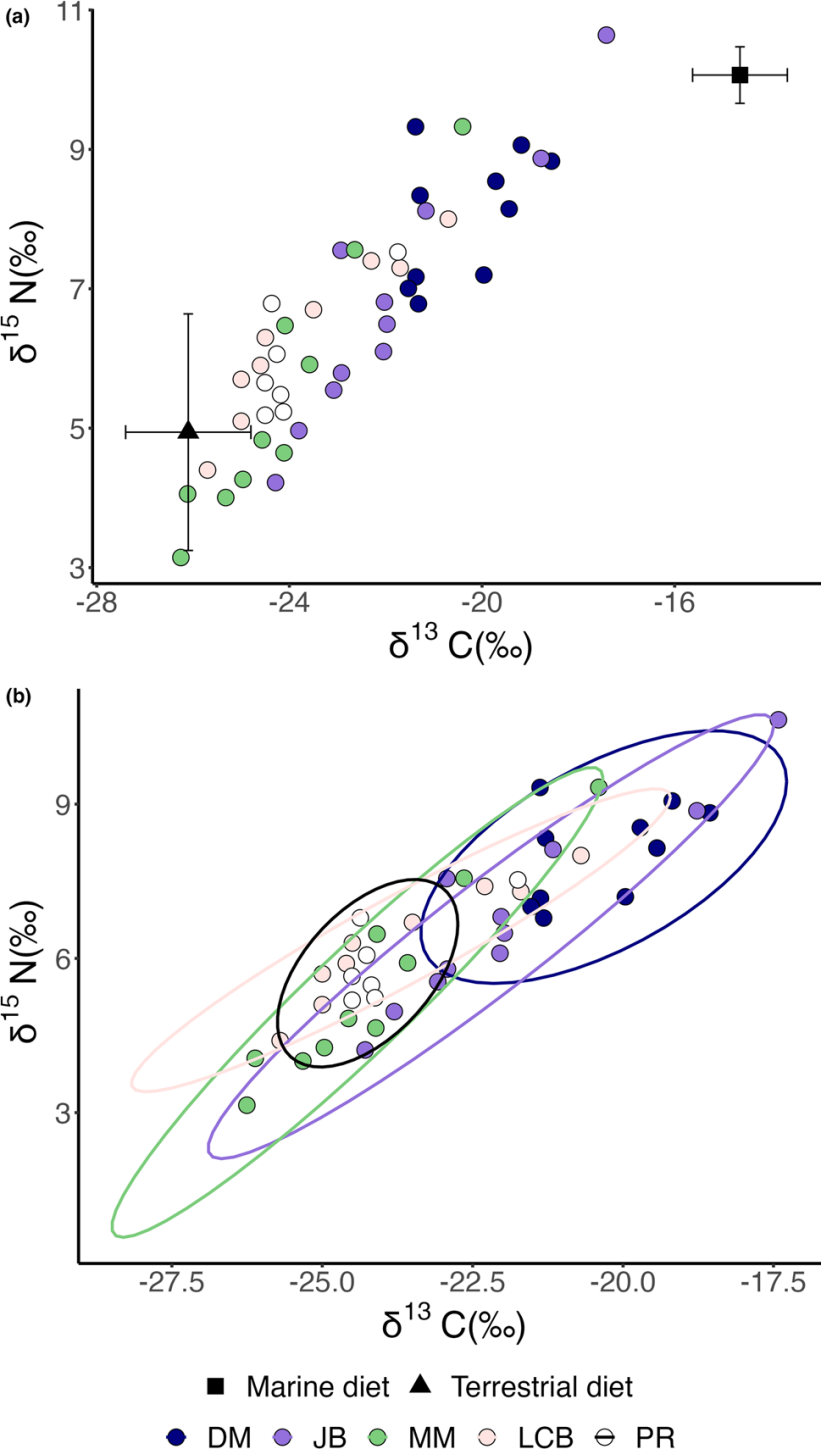
**a** δ^13^C and δ^15^N values of *S. occidentalis* lizards at five Puget Sound beach populations in comparison to δ^13^C and δ^15^N values for marine (*n* = 9) and terrestrial (*n* = 8) diet sources (± standard deviation), adjusted for diet to tissue discrimination values (Lattanzio and Miles 2016) and **b** with isotopic niche spaces indicated by colored ellipses. Ellipses were estimated with the Stable Isotope Bayesian Ellipses in R (SIBER) package

The δ^13^C and δ^15^N values for *S. occidentalis* individuals from all five Puget Sound beach sites fell along a clear mixing line between terrestrial and marine arthropod prey sources (Fig. 3a). There were differences among beach sites; for example, the DM population mean δ^13^C and δ^15^N values were 3.8 and 2.6‰, respectively, higher than the MMP population. The beach sites with the most and least variable δ^13^C and δ^15^N values also had the highest and lowest SEA_C_ (JB = 4.8, and PR = 2.2, respectively) (Fig. 3b).

### Marine diet and isotopic niches of beach lizards

First, we examined how our MixSIAR models differed when using claw versus skin Δ^13^C_tissue-diet_ values. Across all beach lizards, the estimates of percent marine diet using the skin Δ^13^C_tissue-diet_ value were on average 12% higher (range = 4–19% higher) than the estimates using the claw Δ^13^C_tissue-diet_ value. Second, we compared the estimated percent marine diet generated from the MixSIAR model (claw keratin) and the α value equation. At the individual level, the difference ranged from 0 to 31%, and averaged 6%. At the population level, mean differences across beaches ranged 0–11% (Table 1). Moving forward, we only present and interpret percent marine diet calculated from MixSIAR models based on the claw keratin Δ^13^C_tissue-diet_ and Δ^15^N_tissue-diet_ values.

**Table﻿ 1:** The mean (± standard deviation) percent marine diet estimated by MixSIAR and the α equation, the isotopic niche space (SEA_C_), and the average transition zone hillslope for Puget Sound beach populations of *S. occidentalis*

Site	Number of Individuals	Marine diet (%)		SEA_C_	Transition zone slope
		MixSIAR	α		
Des Moines (DM)	10	51 ± 9	40 ± 6	3.20	0.44 ± 0.09
Joemma Beach (JB)	11	39 ± 21	32 ± 11	4.80	0.29 ± 0.11
Lower Chambers Bay (LCB)	9	25 ± 14	23 ± 9	2.31	0.07 ± 0.01
Maury Marine Park (MMP)	10	20 ± 14	20 ± 9	3.41	0.17 ± 0.08
Point Robinson (PR)	7	21 ± 9	21 ± 5	2.25	0.05 ± 0.02

**Fig. 4:**
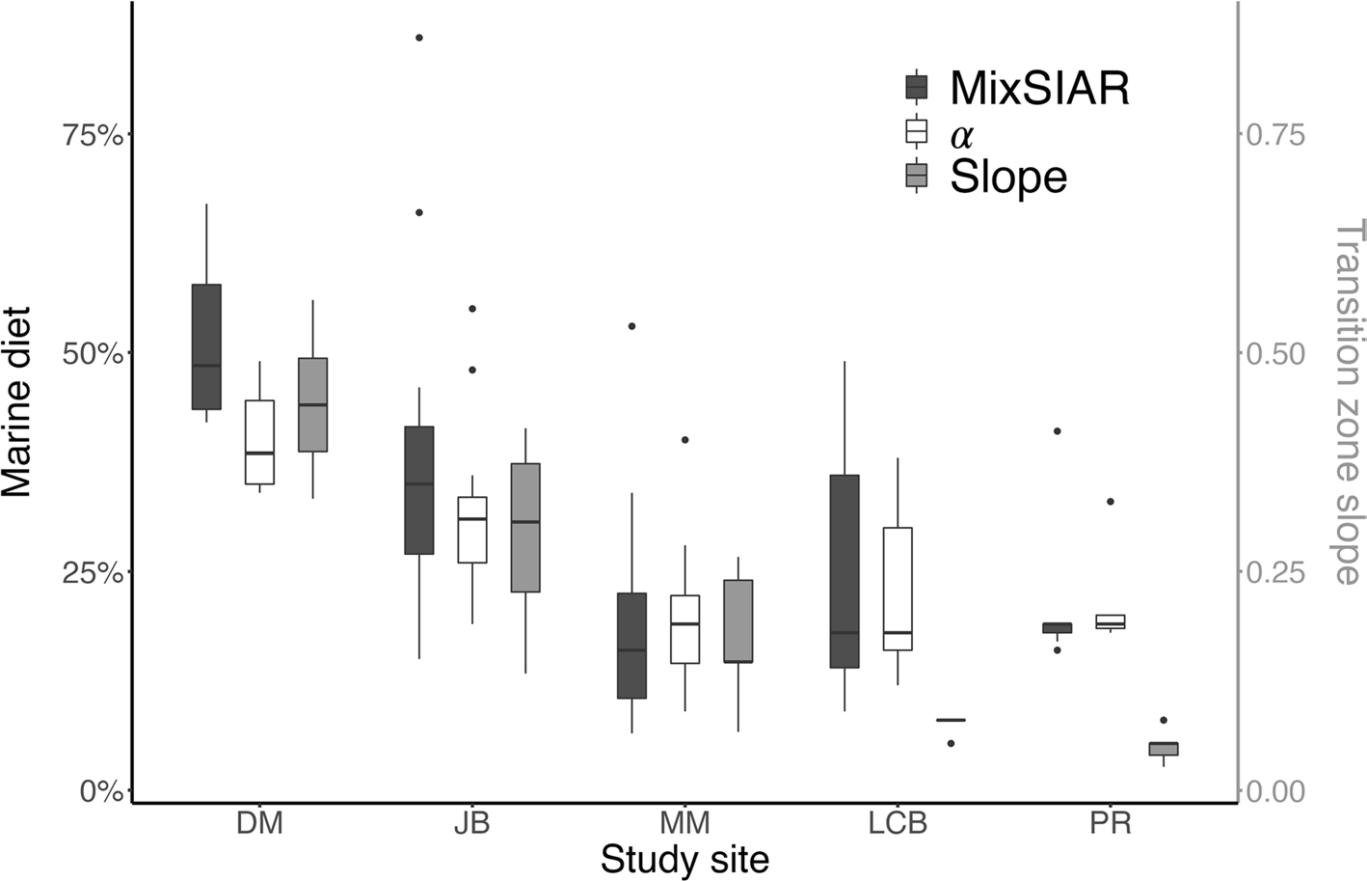
The percentage of diet derived from marine resources for *S. occidentalis* lizards at five Puget Sound beach populations, in addition to the average hillslope of the transition zone between marine and terrestrial environments at beach sites (gray). Percent marine diet was estimated using MixSIAR (black), and α calculation (white)

**Fig. 5:**
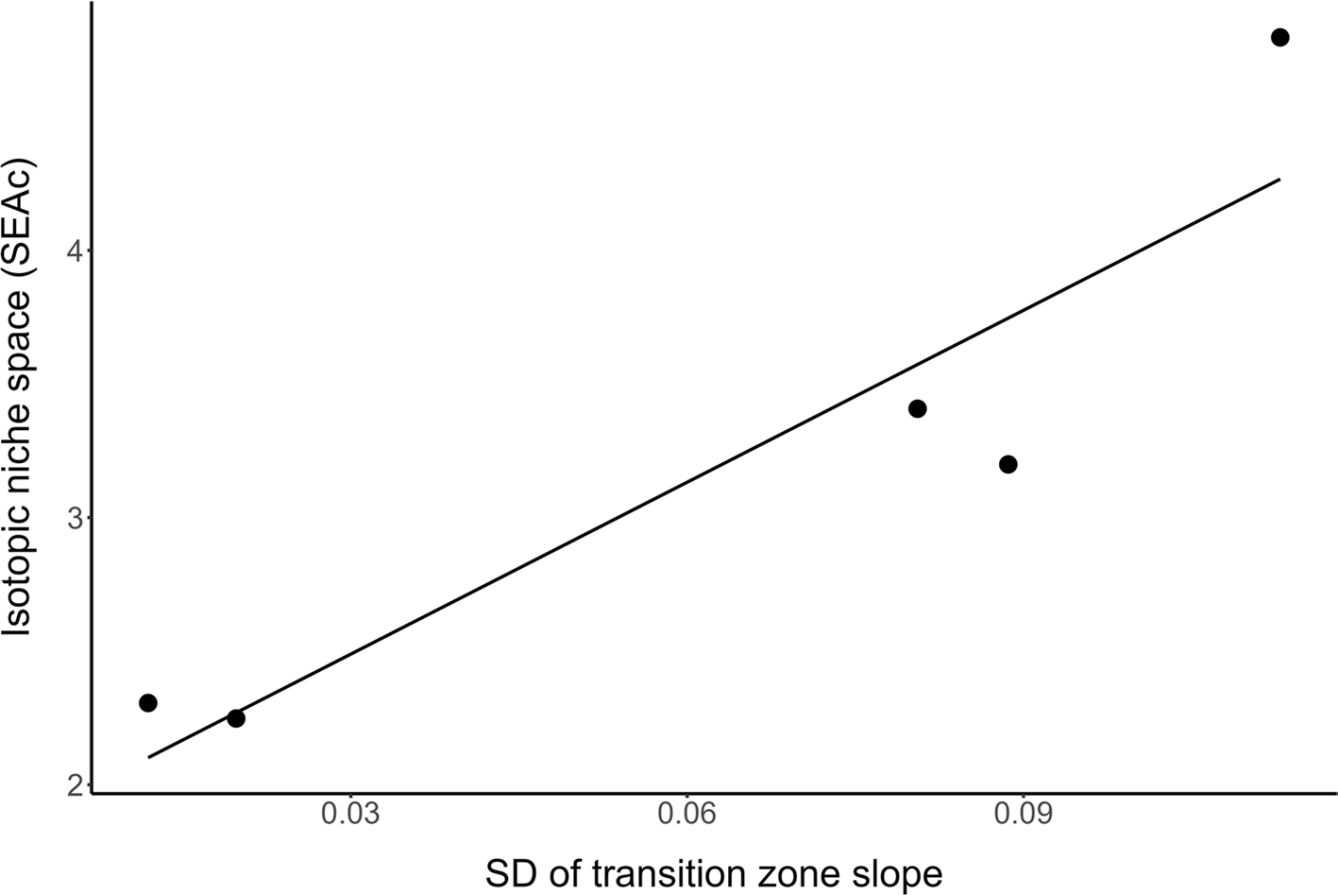
Isotopic niche space for five Puget Sound beach *S. occidentalis* lizard populations estimated by the standard ellipse area (SEA_c_), in relation to the standard deviation (SD) of beach transition zone slope measurements. The SEA_c_ increased significantly with more variable beach topography (*y* = 1.85 + 21.45*x*, *F* = 16.81, df = 1, 3, *P* = 0.026, *R*^2^ = 0.85)

Among individual beach lizards, the percent marine diet ranged from 7 to 86% (Table 1). The average contribution of marine prey varied among beach sites, with a high of 51 ± 9% at DM and a low of 20 ± 14% at MMP (Table 1). There was a positive relationship between average percent marine diet and the average slope of the transition zone between marine and terrestrial environments (*F* = 19.2, df = 1,3, *P* = 0.022, *R*^2^ = 0.87; Fig. 4). For example, lizard populations with the highest and lowest marine diet contributions also had the highest and lowest average transition zone slopes (DM = 44 ± 8.9%, MMP = 17 ± 8.1%). At some beaches (like JB), transition zone slopes across five transects were quite variable, being flat in some locations and steep in others, while at other beaches (like LCB) transition zone, slopes were consistently flat. We predicted that, across beaches, increased variation in transition zone slopes would be associated with broader isotopic niches. Indeed, this relationship was supported, as transition zone slope standard deviation was positively related to SEA_C_, a proxy for isotopic niche space (*F* = 16.81, df = 1,3, *P* = 0.026, *R*^2^ = 0.85, Fig. 5); however, caution in interpretation is needed given the small sample of beaches included in this analysis (*n* = 5).

At an individual level, percent marine diet (log-transformed to meet assumption of normality) did not differ between sexes (*t* = 1.03, df = 45, *P* = 0.308) and did not relate to body condition (*r* = 0.130, df = 45, *P* = 0.384), but tended to positively correlate with both body size (*r* = 0.25, df = 45, *P* = 0.084) and body mass (*r* = 0.29, df = 45, *P* = 0.052).

## Discussion

*S. occidentalis* has a wide distribution across the western United States which encompasses a remarkable breadth of habitats. Our results shed light on the unique ecology of beach-dwelling populations at the northwestern-most extent of the species range, in the South Puget Sound region of Washington state. The diets of Puget Sound beach lizards include both terrestrial and marine prey, which is in contrast to the terrestrial diets of coastal and inland forest lizards. The topography of the marine to terrestrial transition zone may influence marine resource utilization by beach lizards and promote intrapopulation niche diversification.

The δ^13^C and δ^15^N values of Washington lizards were different among the three habitat types. Lizards at the coastal and inland forest habitats had relatively low isotopic values indicative of diets limited to terrestrial food webs (Estupiñán-Montaño et al. 2022). There was a notable difference in lizard δ^13^C values between the coastal and inland forest habitats, which likely reflects hydroclimate rather than diet. C_3_ plant δ^13^C values are inversely correlated with mean annual precipitation (Kohn 2010), and our coastal forest site receives 996 mm/yr compared to 209 mm/yr at the inland forest site (Weather Averages, Washington, USA 2022). We analyzed the most common and abundant grasses at the inland site to confirm that the higher lizard δ^13^C values there were not due to the influence of C_4_ grass resources on diet; all of the grasses were C_3_. The δ^13^C and δ^15^N values of the five beach site lizard populations were on average higher and more variable among individuals than were the values of the forest habitat populations (Fig. 3a). When plotted together with estimates of terrestrial and marine prey, the beach lizard δ^13^C and δ^15^N values for all five sites defined a clear mixing line between the two diet sources. The beach lizard isotope values can be explained by a diet that includes terrestrial and marine prey, and the variability among individuals suggests multiple terrestrial-based, marine-based, and mixed foraging strategies. We highlight that this dietary ecology is unique to the beach sites, and is not observed in the adjacent coastal forest population despite its close proximity to the coastline (< 1000 m), suggesting a direct marine resource subsidy, with the consumption of marine-based prey by beach lizards in the intertidal zone.

The strong linear correlation of δ^13^C and δ^15^N values of our beach lizard dataset was particularly suited for a straightforward thought exercise that is rarely done: the direct comparison of MixSIAR and α value equation approaches to estimating marine diet contribution. For our data, the two approaches yielded similar average estimates of marine contribution and thus similar ecologically relevant understandings of lizard diets for beach populations when MixSIAR estimated percent marine diet below ~ 25%. The α value began to underestimate marine contribution relative to MixSIAR at beaches above the ~ 25% threshold (Table 1). The α value is routinely calculated as a variable in equations for trophic position or trophic chain length in consumers with access to marine diet sources (Post 2002; Pringle et al. 2019; Spiller et al. 2010), but is not presented as a dietary variable itself since the equation relies on only δ^13^C averages (with no associated uncertainty) and thus oversimplifies food web interactions.

The MixSIAR diet source estimates complemented results of the SIBER modeling of isotopic niche for beach lizards, and when considered together allowed us to infer both individual and population levels of dietary variability. Within-beach sites, individual lizards varied in their balance of marine and terrestrial resource use. For example, at JB, one lizard derived 15% of its diet from marine resources while another had 86% marine resource consumption, demonstrating resource use specialization in response to a marine resource subsidy. Other studies have also documented niche and dietary variation of generalist predators subsidized by marine nutrients (Darimont et al. 2009; Davidson et al. 2021). One possible explanation for this individual-level variation in resource use could be demographic factors; however, the percent marine diet of the lizards did not differ by sex or relate to body condition and was correlated only non-significantly with body size and mass. Thus, our results suggest non-demographic factors, such as nearshore geomorphology, are driving lizards within a population to utilize distinct resources in the same environment. Here, we find that beaches with more variable transition zone slopes also have bigger isotopic niche spaces (SEA_C_) (Fig. 5). Though based on only 5 beaches, this pattern suggests that more variable geomorphology promotes intrapopulation niche specialization and more variable marine and terrestrial resource use within populations. Further research with larger sample sizes would increase confidence in this interpretation.

The relationship between geomorphology and utilization of the spatial subsidy is also demonstrated by across-beach patterns. There were substantial differences among beach populations in respect to marine diet contribution and transition zone slope. On average, the beach sites with the highest average marine diet contributions were backed by the steepest topographic features whereas beaches sites with lower dependence on marine prey were backed by low transition zone slopes (Fig. 4). This is consistent with previous studies that have shown accessibility between adjacent ecosystems (via physical habitat structure and geology) can enhance or restrict nutrient flow between food webs and dictates predator–prey interactions (Briggs et al. 2012; Dolson et al. 2009; Pe’er et al. 2006; Ryan et al. 2013). While it is possible that the abundance of wrack, and thus the availability of algae-consuming arthropod prey for lizards, contributes to variation in marine diet among beach populations, we controlled for differences in physical characteristics that may regulate wrack abundance (e.g., sand and gravel substrate, low angle beach slope, and southerly exposure) by selecting study sites with little variation in these characteristics (Obrist et al. 2022; Wickham et al. 2020). Anecdotally, we observed that wrack was present at all sites during field sampling, and we used Google Earth historical satellite imagery to confirm the persistence of wrack lines at the study sites from 2010 to 2022 (Google Earth Pro 2022).

Marine preys were consumed to some extent by all Puget Sound beach lizards highlighting the ubiquity of this resource subsidy which may support species’ success at the northwestern range limit of *S. occidentalis*. The subsidy may also explain the fragmented and restricted distribution of *S. occidentalis* in the Puget Sound region compared to the continuous distributions of southern populations and their significantly greater abundance on beach habitats compared to non-subsidized near-coastal and inland regions (Bouzid et al. 2022; Davis et al. 2022). Marine-subsidized diets offer a potential mechanism for lizard populations to overcome otherwise range-limiting factors, such as nutrient and prey limitations (Estupiñán-Montaño et al. 2022), which merits further investigation.

An ecological interaction that is dependent on coastal habitat and on marine resource availability via sea wrack is particularly susceptible to anthropogenic disruption by changes in sea level and ocean conditions (Hyndes et al. 2022). As we have shown here, lizards facilitate the flow of resources between marine and terrestrial habitats, providing another example of ecosystem services provided by reptiles (Valencia-Aguilar et al. 2013). This dynamic relies on the existence of suitable beach habitat. Puget Sound beach *S. occidentalis* rely on driftwood substrate at the high tide line for basking and foraging, and adjacent vegetated hillsides for nesting habitat. These habitats are especially vulnerable to development in the Puget Sound region, including beach armoring (sea walls) which reduces driftwood habitat and seaweed deposition on beaches and subsequent intertidal arthropods on which the lizards feed, potentially disrupting the strength of the resource subsidy (Dethier et al. 2016; Jaramillo et al. 2021). In addition to immediate threats of shoreline development, long-term climate change implications include rising sea levels and water temperatures in the Puget Sound (Snover et al. 2005). Sea level and temperature changes can lead to the loss of nearshore habitats, and may lead to changes in nutrient availability by altering overall macroalgae abundance and thus arthropod prey items, threatening Puget Sound *S. occidentalis* beach populations (Snover et al. 2005).

Our study documented a marine-terrestrial spatial subsidy enriching the diets of *S. occidentalis* populations at their northwestern range limit, on Puget Sound beaches. Coastal forest (1000 m from beach site) and inland (central WA) *S. occidentalis* populations occupied distinct bivariate isospace compared to beach populations, emphasizing the novelty of the marine resource utilization of beach lizards. Beach sites with the steepest average transition zone slope were home to lizards with the highest marine diet contributions, suggesting that topography is an important factor in mediating the strength of fine-scale spatial subsidies. Individual-level resource use variation of beach *S. occidentalis* showed that some lizards within a single population depended more heavily on marine resources, while others had almost exclusive terrestrial diets. Intrapopulation level variation was also correlated with the variability of the beach topography, providing evidence that landscape heterogeneity promotes niche specialization in the presence of a marine spatial subsidy. This raises questions about individual specialization, perhaps based on body size, and niche variation within generalist consumer populations in response to landscape characteristics. Lastly, urban beach development and rising sea levels are continual threats to beach habitat of *S. occidentalis* while Puget Sound water temperatures may impact prey availability of lizards by altering macroalgae and intertidal arthropod prey item abundances.

## Data Availability

Data will be available on Dryad following manuscript acceptance. https://datadryad.org/stash/share/FI3Cg6rHScN-LxU9JPl_nEqneJH6Ptk1FrMShcCBDjE
